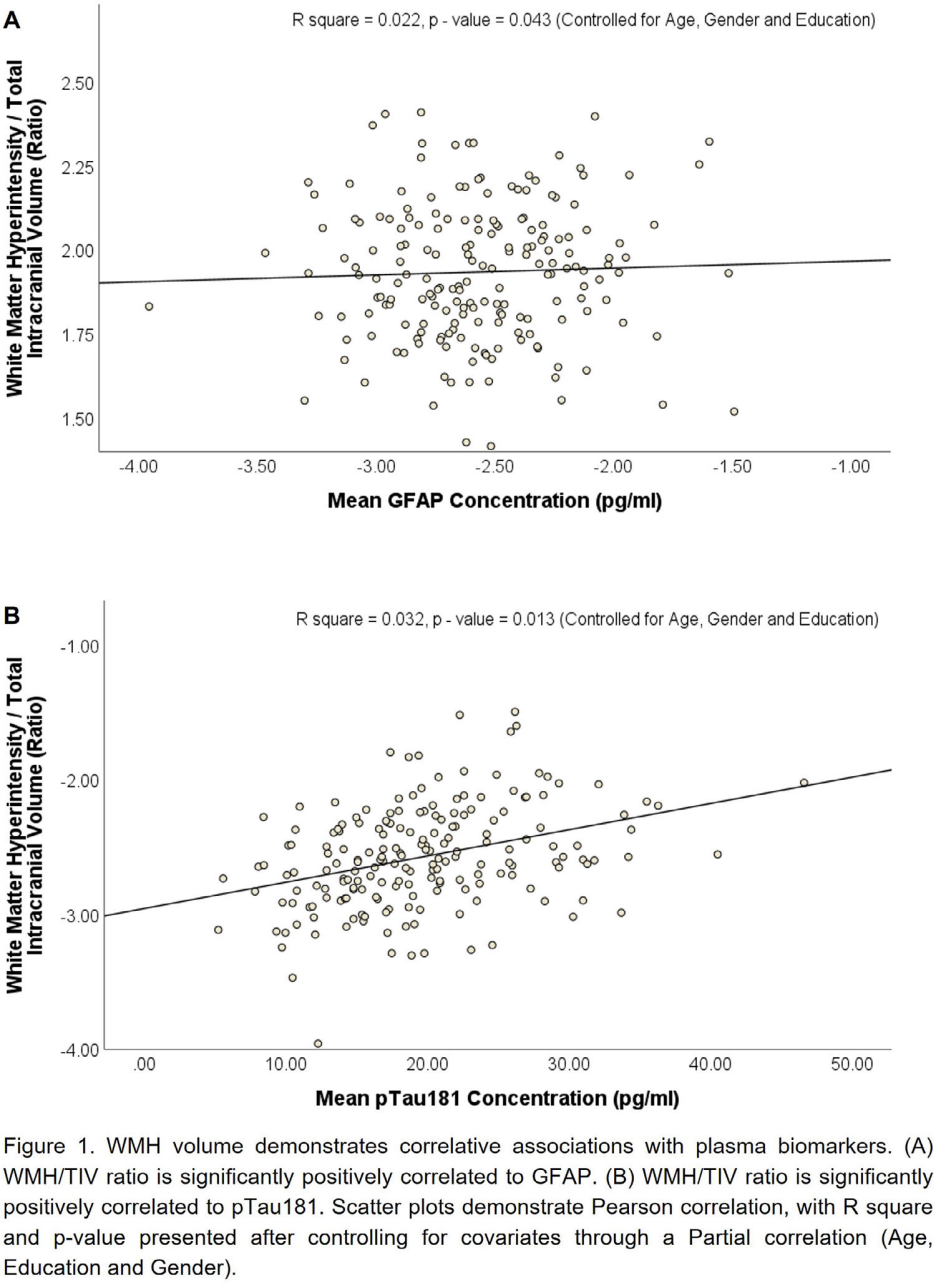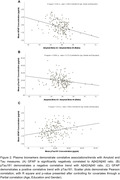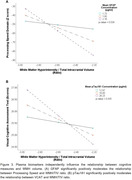# Plasma GFAP and pTau‐181 Moderate the Relationship between Cognition and White Matter Hyperintensities in a Southeast Asian Non‐Demented‐at‐Risk Cohort

**DOI:** 10.1002/alz.091177

**Published:** 2025-01-09

**Authors:** Gurveen Kaur Sandhu, Ashwati Vipin, Fatin Zahra Zailan, Pricilia Tanoto, Faith Phemie Hui En Lee, See Ann Soo, Smriti Ghildiyal, Shan Yao Liew, Isabelle Yu Zhen Tan, Yi Jin Leow, Dilip Kumar, Mohammed Adnan Azam, Wayne Freeman Chong, Nagaendran Kandiah

**Affiliations:** ^1^ Lee Kong Chian School of Medicine, Nanyang Technological University, Singapore Singapore

## Abstract

**Background:**

Astrocyte reactivity marked by elevations in Glial Fibrillary Acidic Protein(GFAP), was found to increase Phosphorylated Tau181(pTau181) induced neurodegenerative effects in Alzheimer’s Disease (Bellaver et al., 2023). Nevertheless, the effect of the GFAP‐ pTau181 axis in vascular/mixed dementias has yet to be completely elucidated. In this study, we investigated the effect of GFAP‐pTau181 on cognition in a Southeast Asian cohort having high burden of cerebrovascular disease.

**Methods:**

A cross‐sectional study was conducted in 190 non‐demented but at risk, Southeast Asian community participants recruited into the Biomarker and Cognition Study. Neuropsychological Assessments of global cognition [Visual Cognitive Assessment Test(VCAT)] and processing speed were measured (Kumar, 2018; Nagaendran et al., 2016; Tian et al., 2022; Wiseman et al., 2018). The burden of white matter hyperintensities (WMH), a surrogate measure for cerebral small vessel disease was quantified using the automated lesion growth algorithm (Lesion Segmentation Tool) (Vipin et al., 2021). Plasma biomarkers (GFAP, Aβ40, Aβ42, pTau181) were quantified using the Single Molecule Array(Simoa) platform (Wilson et al., 2016). A partial correlation analysis was used to demonstrate associations between variables. A moderation analysis was conducted to determine if pTau/GFAP influenced the relationship between cognition and WMH, via linear regression analysis with the inclusion of interaction terms (WMH*pTau/GFAP).

**Results:**

Findings indicate that, WMH volume positively correlated to GFAP and pTau181(Figure 1A and 1B). Information processing speed and VCAT negatively correlated to WMH volume. GFAP negatively correlated to Aβ42/Aβ40 ratio (Figure 2A). pTau181 demonstrated a negative correlative trend with Aβ42/Aβ40 ratio (Figure 2B) and a positive correlative trend with GFAP (Figure 2C). Moderation analysis revealed that GFAP and pTau181 independently influenced the relationship between cognitive measures and WMH volume, such that increasing WMH volume and GFAP/pTau181 levels related to deteriorating processing speed and VCAT scores (Figure 3A and 3B).

**Conclusion:**

GFAP, a neuroinflammatory measure moderates the relationship between WMH and cognition and may be a useful biomarker for the detection and prognostication of cognitive impairment among Southeast Asians. The pathobiology could be a result of early upstream astrocyte mediated modulation of Blood‐Brain Barrier permeability and resultant perfusion dynamics. pTau181 facilitated effects are likely downstream events of GFAP activation. Further mechanistic validation in a larger longitudinal study is underway.